# Hypnotised by Your Phone? Smartphone Addiction Correlates With Hypnotisability

**DOI:** 10.3389/fpsyt.2020.00578

**Published:** 2020-06-25

**Authors:** Jay A. Olson, Moriah Stendel, Samuel Veissière

**Affiliations:** ^1^ Department of Psychiatry, McGill University, Montreal, QC, Canada; ^2^ Department of Psychology, University of Oregon, Eugene, OR, United States; ^3^ Culture, Mind, and Brain Program, McGill University, Montreal, QC, Canada; ^4^ Department of Anthropology, McGill University, Montreal, QC, Canada

**Keywords:** problematic smartphone use, smartphone addiction, technology addiction, hypnosis, hypnotisability

## Abstract

Hypnosis and heavy smartphone use are both characterised by absorbed states in which one loses track of time and responds automatically to stimuli. In this pre-registered study, we tested whether there was a relationship between smartphone addiction and hypnotisability: one’s tendency to follow suggestions under hypnosis. Over 11 public lectures, we hypnotised 641 student-aged participants; after the hypnosis session, participants completed the Smartphone Addiction Scale (Short Version). There was a positive correlation between hypnotisability and smartphone addiction (*r* = .17, 95% CI [.09, .24], *p* < .001) with a magnitude similar to standard predictors of hypnotisability. This correlation was small but unlikely spurious: it was positive in 10 of the 11 samples (including two from psychology courses) and persisted in a follow-up several months later. The addiction scores in this Canadian sample were unexpectedly high (*M* = 31.41) compared to other countries. We hypothesise that targeting the absorbed, time-distorted, and automatic use of smartphones may promote healthier phone habits.

## Introduction****

Smartphone use has risen dramatically in the past decade. In the United States, 96% of young adults own a smartphone (1) and half of teenagers report feeling addicted to their phones (2); other developed countries show similar rates (3). Researchers and reporters have compared this heavy phone use to a trance or hypnosis (4, 5). Madrigal (6) even likens the “hypnotic” state of endless social media scrolling to the trance-like absorption of slot machines (7), due to their intermittent rewards (8). If heavy smartphone use can resemble hypnosis, people who are more hypnotisable may also be more prone to *problematic smartphone use*, in which phone use interferes with daily life (9). No studies have yet attempted to link these phenomena, so we present the first test of this hypothesis.

The American Psychological Association defines hypnosis as a “state of consciousness involving focused attention and reduced peripheral awareness characterised by an enhanced capacity for response to suggestion” (10), though researchers debate aspects of this definition (11). We propose that hypnosis and heavy smartphone use may share phenomenological features such as absorption, time distortion, and automaticity. Absorption refers to the tendency to become immersed in one’s thoughts or experiences (12), such as forgetting about the movie theatre while watching a film. Absorption predicts addictive behaviours in the context of gambling (7, 13), video games (14), internet use (15), and problematic smartphone use (16). Heavy smartphone users often find themselves in these absorbed states, leading to the term “smartphone zombie” to describe the head-down phone-absorbed user, who occasionally walks into other pedestrians—or into traffic (17). Several cities have already established special walking lanes for smartphone users, and researchers have developed phone functions to warn users about incoming objects in the environment, highlighting the extent of absorbed attention when using a phone. Similarly, many people report being heavily absorbed in their experience when under hypnosis (18, 19), and trait absorption tends to correlate with hypnotisability (12, 20, 21).

Hypnosis and smartphone use can also both distort time perception. People underestimate the amount of time spent on their phone (22), with heavier smartphone users showing greater distortions (23). Accordingly, several measures of problematic smartphone use probe whether people use their phone longer than they intend (24, 25). Similar time distortions are well known in hypnosis; people consistently underestimate how long they were hypnotised for, and the higher their hypnotisability, the larger this distortion (26, 27).

Finally, hypnosis and smartphone use can both elicit automatic behaviours with a reduced feeling of control. People can become side-tracked while simply trying to check the time on their phone (28) and report being “sucked down a rabbit hole of un-productivity” (29) or “into some mindless … black hole” (30). People commonly report a loss of self-control when using their phones (30), especially if they feel addicted to them (31). Relatedly, people feel less control over their behaviours under hypnosis (32), such as feeling their arm lifting without their apparent control. People can even engage in complex behaviours, such as writing sentences with a pen, without feeling like they are controlling their actions (33).

Given these phenomenological similarities, we hypothesised that people who are more hypnotisable—those more likely to follow suggestions under hypnosis (10)—would be more prone to problematic smartphone use. We anticipated a correlation around *r* = .19, similar to other predictors of hypnotisability (34, 35).

## Materials and Methods

### Procedure

We held 11 public lectures on hypnosis at McGill University in Montreal, Canada. Two of these lectures were for introductory psychology courses. After a 45-min lecture, we invited the audience to participate in a study, during which we administered standard measures of hypnotisability and problematic smartphone use. Each lecture, almost everyone stayed to participate without compensation, so there was no further selection bias. The protocol was approved by the McGill University Research Ethics Board (#338-0117).

### Participants

In total, 718 participants completed the study. We excluded those without smartphones (*n =* 40) or with missing values on the hypnosis (*n* = 22) or smartphone questionnaires (*n* = 15). (Imputing these missing values using mean substitution would have changed no decisions about our hypotheses.) After the exclusions, 641 participants remained; the majority were women (71%) and the average age was 21.2 (*SD* = 3.6, range: 18 to 47).

### Measures

#### Hypnotisability

After consenting to the study, participants completed the Harvard Group Scale of Hypnotic Susceptibility Form A (36), the most common scale of hypnotisability. This procedure has two parts. First, the experimenter plays a standard 45-min audio recording of a hypnotic induction (e.g., “Your eyelids are getting heavy…”) followed by a series of 12 verbal suggestions. For example, the recording suggests that the participant’s head will fall forward or that they will be momentarily unable to open their eyes. Second, after the suggestions, the recording leads participants out of hypnosis; they then complete a questionnaire reporting how many of the 12 suggestions they successfully followed. Higher scores indicate greater hypnotisability. The scale has good internal consistency in previous samples from the same city (KR-20 = .84) (37), but it was lower in our sample (.64). We considered the reliability sufficient for this preliminary research (38).

#### Problematic Smartphone Use

Participants then completed the Smartphone Addiction Scale (Short Version) (25), the most common measure of problematic smartphone use. This scale quantifies how much smartphones interfere with daily life; we are agnostic about whether this constitutes an addiction in the general population (39). An example item is: “I feel impatient and fretful when I am not holding my smartphone”. We made minor changes to the wording of some of the questions to fix grammatical issues and improve clarity for our sample (see **Appendix A**). The 10 items use Likert scales ranging from “Strongly disagree” (1) to “Strongly agree” (6), for a total score between 10 and 60. Higher scores indicate a greater risk of addiction as judged by clinicians (25, 40). The scale usually has high internal consistency (Cronbach’s *α* = .91) (25) which was similar in our sample (.83). To assess test-retest reliability, an exploratory subsample of the participants (*n* = 54) retook the Smartphone Addiction Scale approximately 6 months later (*M* = 185.5 days, *SD* = 178.3, range: 3 to 535) in an unrelated study. Beyond demographics, no other measures were collected.

### Analysis

All aspects of the study and analysis were pre-registered online (see https://osf.io/juk4n). Using linear regression, we tested whether hypnotisability predicted smartphone use (partial model) before adding sex as an additional predictor (full model). We anticipated a small correlation which would require 300 valid data points for 90% statistical power. We continued to hold public lectures until we reached this number.

We then replicated these results using an identical procedure. We describe both samples together and focus on correlations and robust standardised mean differences signified as *d_R_* (41). The regression results (**Appendix B**) are confirmatory and all other tests are exploratory. All assumptions for the tests were reasonable; hypnotisability (42) and problematic smartphone use are often normally distributed (43). Square brackets denote bootstrapped 95% confidence intervals (44).

## Results and Discussion

### Hypnotisability Predicted Problematic Smartphone Use

**Figure 1:**
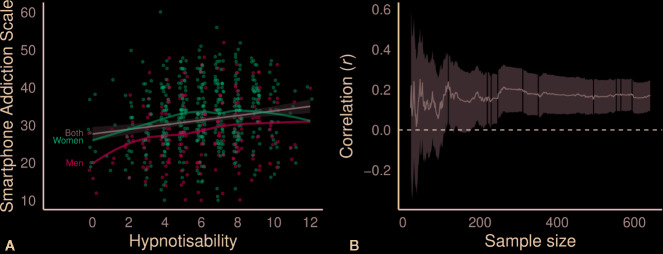
**(A)** Correlation between problematic smartphone use and hypnotisability **(B)** across the sessions. In **(A)**, curved lines show smoothed averages for each sex, straight line shows linear prediction, band shows 95% confidence interval, and (jittered) dots represent participants. In **(B)**, line shows correlation coefficient across participants, band shows 95% confidence interval, and white lines demarcate sessions.

Scores on the Smartphone Addiction Scale positively correlated with the number of hypnotic suggestions that participants followed (*r*(639) = .17 [.09, .24], *p* < .001; **Figure 1A**). The correlation was small, as expected in our pre-registration (*r* = .186), but it was fairly stable (**Figure 1B**) given the large sample size (45). Indeed, the sample correlations were in the positive direction for 10 of the 11 public lectures. The correlation was unlikely due to selection bias; we also saw a positive correlation in the two samples taken from psychology courses (*r* = .29 [.11, .44]). Hypnotisability has few strong predictors, so small correlations are common; traits such as the Big Five show correlations with hypnotisability between .01 and .19 (34, 35). There were roughly linear relationships for men (*r*(186) = .21 [.08, .33], *p* = .004) and women (*r*(449) = .15 [.06, .24], *p* = .001). **Table B1** shows the regression results for each sample.

The average hypnotisability score was 6.12 [5.93, 6.32], with little difference between men (6.10 [5.73, 6.45]) and women (6.13 [5.93, 6.34]; *d_R_* = 0.02 [–0.02, 0.20]). These averages resembled previous samples from the same city (37, 46).

In some studies, predictors of hypnotisability are inflated when completing other measures in the same context as the hypnosis (20, 21). This was unlikely here, since our test-retest sample showed a similar correlation six months later in a different context (*r* = .21 [−.08, .46], excluding one participant with a difference score of *z* = 4.16). The test-retest reliability of the smartphone measure was high (*r* = .78 [.62, .87]) across participants with the full range of hypnotisability scores (i.e., 0 to 12).

### Problematic Smartphone Use Was High

The average Smartphone Addiction Scale (Short Version) score was 31.41 [30.68, 32.10]. Women scored 32.62 [31.82, 33.42] and men scored 28.48 [27.13, 29.69] (*d_R_* = 0.43 [0.15, 0.75]). Using the scale authors’ criteria (40), 51% of the women and 39% of the men would have a high risk of phone addiction.

These scores from Montreal, Canada were unexpectedly high. Our average was higher than in samples from Spain (21.10) (47), Germany (23.09) (48), Switzerland (23.45) (49), Belgium (24.00) (47), Romania (24.2) (50), and the midwestern United States (27.01) (51), but it was similar to adolescent samples in Turkey (31.37) (52), and China (34.0) (53). The reason for our high scores is unclear. It is unlikely that our minor rewording of the questionnaire items had a large effect, given that the scale has been translated into several languages without apparent inflation of the scores. Further, selection bias cannot entirely explain these findings; our scores resemble those obtained in unrelated studies we are conducting in the same city. The field may benefit from a comprehensive review of problematic smartphone use scores across countries to help explain these regional differences (3).

### Limitations and Future Studies

Our study had several limitations. First, all measures were self-reported, as is common when measuring hypnosis and problematic smartphone use. Future studies adding objective measures such as screen time tracking could reveal whether hypnotisable participants use their phones more, especially for absorbing activities such as gaming or social media. Second, our sample was young (primary 18 to 22 years old), so we can only generalise to the student population but not to older adults. Since problematic smartphone use primarily affects youth (40), though, the age of our sample was appropriate. Third, given our correlational design we could not assess causality or the direction of the relationship. It seems unlikely that phone use affected hypnotisability, since hypnotisability is generally stable across adulthood (54). We thus expect that either hypnotisability affects smartphone use, or an underlying construct is acting as a third variable. One candidate may be dissociation, in which one disengages from the sense of self or the environment (55, 56). Similar to absorption, dissociative experiences predict problematic technology use (16, 57). Dissociation is related to hypnotisability in some highly hypnotisable participants (58), but it generally shows inconsistent correlations (59) so cannot account for all of our results. Another possible candidate could be sociality. Some theories posit that hypnosis is primarily a social context involving a set of expectations about what will occur (60), such as the belief that one will automatically follow the hypnotist’s suggestions. Hypnotisability may also relate to responsiveness to social cues more generally (61, 62). Relatedly, using phones for social purposes predicts habitual use and addictive behaviour (63, 64). Future studies could test whether dissociation, absorption, or sociality could be the third variable underlying the relationship.

Our findings may also point towards potential interventions. If the positive correlation here reflects the phenomenological similarities between hypnosis and problematic smartphone use (i.e., absorption, time distortion, and automaticity), interventions could target these components. To reduce automatic interactions, behavioural interventions could reduce the salience of the phone or make it more effortful to use (65), for example by keeping the phone further out of reach (66) or limiting sporadic notifications (67). Indeed, combining similar strategies can effectively reduce problematic smartphone use (Olson et al., in preparation).

## Conclusion

In the current “attention economy”, smartphone use translates into data collection and advertising revenue, giving developers economic incentive to keep users absorbed (68). As digital interfaces continue to become more immersive, so too may users’ absorption, time distortion, and automatic behaviour. The relationship between hypnotisability and problematic smartphone use may thus continue to strengthen, further necessitating interventions to tackle these components.

## Data Availability Statement

The full data set is available on the Open Science Framework (https://osf.io/etyj6/).

## Ethics Statement

This study was reviewed and approved by the McGill University Research Ethics Board (#338-0117). The participants provided their written informed consent to participate in this study.

## Author Contributions 

JO led all aspects of the study. MS assisted with data collection. SV provided funding and supervised the study. All authors contributed to the article and approved the submitted version.

## Conflict of Interest

The authors declare that the research was conducted in the absence of any commercial or financial relationships that could be construed as a potential conflict of interest.

## Appendix A Smartphone Addiction Scale—Short Version (SAS-SV)

We made minor changes to the wording of the SAS-SV items to fix grammatical issues, improve clarity, and update the examples of social networking sites. We confirmed with the scale’s authors that these changes did not impact the intended meaning of the items (Kwon, personal communication, 2019). As in the original measure, each item used a Likert scale from 1 to 6 (strongly disagree, disagree, weakly disagree, weakly agree, and so on). The differences are highlighted in bold below.

1. **I miss work that I planned,** due to smartphone use.
2. **I have** a hard time concentrating in class, while doing assignments, or while working, due to smartphone use.
3. **I feel** pain in my wrists or at the back of my neck while using a smartphone.
4. **I wouldn’t** be able to stand not having a smartphone.
5. **I feel** impatient and fretful when I am not holding my smartphone.
6. **I have** my smartphone on my mind even when I am not using it.
7. **I would** never give up using my smartphone even if my daily life **were** greatly affected by it.
8. **I constantly check** my smartphone so as not to miss conversations between other people on Twitter, Facebook, **Snapchat, Instagram, or other social media**.
9. **I use** my smartphone longer than I intend.
10. **People** around me tell me that I use my smartphone too much.

Original items (40):

1. Missing planned work due to smartphone use.
2. Having a hard time concentrating in class, while doing assignments, or while working due to smartphone use.
3. Feeling pain in the wrists or at the back of the neck while using a smartphone.
4. Won’t be able to stand not having a smartphone.
5. Feeling impatient and fretful when I am not holding my smartphone.
6. Having my smartphone in my mind even when I am not using it.
7. I will never give up using my smartphone even when my daily life is already greatly affected by it.
8. Constantly checking my smartphone so as not to miss conversations between other people on Twitter or Facebook.
9. Using my smartphone longer than I had intended.
10. The people around me tell me that I use my smartphone too much.

## Appendix B Confirmatory tests

**Table B1:** Confirmatory regression results for the pre-registered sample (*n* = 310) and replication (*n* = 331).

Sample	Model	Sex	Predictor	*B*	SE	*t*	*p*
1	Partial	Both	(Intercept)	26.45	1.36		
			Hypnotisability	0.75	0.21	3.57	<.001
		Men	(Intercept)	22.17	2.54		
			Hypnotisability	1.08	0.41	2.64	.010
		Women	(Intercept)	28.79	1.56		
			Hypnotisability	0.55	0.24	2.33	.021
	Full	Both	(Intercept)	24.29	1.49		
			Hypnotisability	0.71	0.21	3.43	<.001
			Sex (F)	3.55	1.09	3.25	.001
2	Partial	Both	(Intercept)	28.91	1.30		
			Hypnotisability	0.47	0.19	2.44	.015
		Men	(Intercept)	25.67	2.07		
			Hypnotisability	0.45	0.29	1.53	.130
		Women	(Intercept)	29.74	1.57		
			Hypnotisability	0.53	0.24	2.24	.026
	Full	Both	(Intercept)	25.31	1.51		
			Hypnotisability	0.51	0.19	2.69	.007
			Sex (F)	4.60	1.04	4.43	<.001

## References

[1] Pew Research Center Mobile fact sheet. In: Pew Research Center: Internet, Science & Tech (2019).

[2] Felt LJ Robb MB Technology addiction: Concern, controversy, and finding balance. In: Common Sense Media (2016). Available at: https://www.commonsensemedia.org/research/technology-addiction-concern-controversy-and-finding-balance.

[3] Fischer-Grote L Kothgassner OD Felnhofer A Risk factors for problematic smartphone use in children and adolescents: A review of existing literature. neuropsychiatrie (2019) 33:179–90. 10.1007/s40211-019-00319-8 PMC6901427 31493233

[4] Colic-Peisker V Flitney A The promise and threat of the internet age. In: The age of post-rationality. Singapore: Palgrave Macmillan (2018). p. 183–211.

[5] Greene N (2018). I tried to cure my smartphone addiction using my smartphone. VICE.

[6] Madrigal AC (2013). The machine zone: This is where you go when you just can't stop looking at pictures on Facebook. The Atlantic. Available at: https://www.theatlantic.com/technology/archive/2013/07/the-machine-zone-this-is-where-you-go-when-you-just-cant-stop-looking-at-pictures-on-facebook/278185/.

[7] Schüll ND Addiction by design: Machine gambling in Las Vegas. Princeton, NJ: Princeton University Press (2014).

[8] Aranda JH Baig S Toward "JOMO": The joy of missing out and the freedom of disconnecting. Proceedings of the 20th international conference on human-computer interaction with mobile devices and services, (2018) 19. 10.1145/3229434.3229468

[9] Panova T Carbonell X Is smartphone addiction really an addiction? J Behav Addict (2018) 7:252–9. 10.1556/2006.7.2018.49 PMC6174603 29895183

[10] Elkins GR Barabasz AF Council JR Spiegel D Advancing research and practice:The revised APA division 30 definition of hypnosis. Int J Clin Exp Hypnosis (2014) 63:1–9. 10.1080/00207144.2014.961870 25365125

[11] Lynn SJ Green JP Kirsch I Capafons A Lilienfeld SO Laurence J-R Grounding hypnosis in science: The “New” APA division 30 definition of hypnosis as a step backward. Am J Clin Hypnosis (2015) 57:390–401. 10.1080/00029157.2015.1011472 25928778

[12] Tellegen A Atkinson G Openness to absorbing and self-altering experiences (“absorption”), a trait related to hypnotic susceptibility. J Abnormal Psychol (1974) 83:268. 10.1037/h0036681 4844914

[13] Rémond J-J Romo L Analysis of gambling in the media related to screens: Immersion as a predictor of excessive use? Int J Environ Res Public Health (2018) 15:58. 10.3390/ijerph15010058 PMC5800157 29301311

[14] Barnes SJ Pressey AD Caught in the web? Addictive behavior in cyberspace and the role of goal-orientation. Technol Forecasting Soc Change (2014) 86:93–109. 10.1016/j.techfore.2013.08.024

[15] Bozoglan B Demirer V Sahin I Problematic internet use: Functions of use, cognitive absorption, and depression. Comput Hum Behav (2014) 37:117–23. 10.1016/j.chb.2014.04.042

[16] De Pasquale C Sciacca F Hichy Z Smartphone addiction and dissociative experience: An investigation in italian adolescents aged between 14 and 19 years. Int J Psychol Behav Anal (2015) 1:109. 10.15344/2455-3867/2015/109

[17] Kim D Han K Sim JS Noh Y Smombie guardian: We watch for potential obstacles while you are walking and conducting smartphone activities. PloS One (2018) 13:e0197050. 10.1371/journal.pone.0197050 29944656 PMC6019093

[18] Kihlstrom JF Register PA Hoyt IP Albright JS Grigorian EM Heindel WC Dispositional correlates of hypnosis: A phenomenological approach. Int J Clin Exp Hypnosis (1989) 37:249–63. 10.1080/00207148908414476 2753575

[19] Nash M Barnier AJ The Oxford Handbook of Hypnosis: Theory, Research, and Practice. Oxford, UK: Oxford University Press (2012).

[20] Council JR Green JP Examining the absorption-hypnotizability link: The roles of acquiescence and consistency motivation. Int J Clin Exp Hypnosis (2004) 52:364–77. 10.1080/00207140490883950 15590504

[21] Milling LS Kirsch I Burgess C Hypnotic suggestibility and absorption: Revisiting the context effect. Contemp Hypnosis (2000) 17:32–41. 10.1002/ch.190

[22] Lee H Ahn H Nguyen TG Choi S-W Kim DJ Comparing the self-report and measured smartphone usage of college students: A pilot study. Psychiatry Invest (2017) 14:198. 10.4306/pi.2017.14.2.198 PMC5355019 28326119

[23] Lin Y-H Lin Y-C Lee Y-H Lin P-H Lin S-H Chang L-R Time distortion associated with smartphone addiction: Identifying smartphone addiction via a mobile application (app). J Psychiatr Res (2015) 65:139–45. 10.1016/j.jpsychires.2015.04.003 25935253

[24] Bianchi A Phillips JG Psychological predictors of problem mobile phone use. CyberPsychol Behav (2005) 8:39–51. 10.1089/cpb.2005.8.39 15738692

[25] Kwon M Lee J-Y Won W-Y Park J-W Min J-A Hahn C Development and validation of a smartphone addiction scale (SAS). PloS One (2013) 8:e56936. 10.1371/journal.pone.0056936 23468893 PMC3584150

[26] Bowers KS Time distortion and hypnotic ability: Underestimating the duration of hypnosis. J Abnormal Psychol (1979) 88:435. 10.1037/0021-843X.88.4.435 479465

[27] Cooper LF Erickson MH Time distortion in hypnosis: An experimental and clinical investigation. 2nd ed. Carmarthen, UK: Crown House Publishing Ltd (2002).

[28] Montag C Kannen C Lachmann B Sariyska R Duke E Reuter M The importance of analogue zeitgebers to reduce digital addictive tendencies in the 21st century. Addictive Behav Rep (2015) 2:23–7. 10.1016/j.abrep.2015.04.002 PMC5845952 29531991

[29] Newport C Digital minimalism: Choosing a focused life in a noisy world. London: Penguin (2019).

[30] Lukoff K Yu C Kientz J Hiniker A (2018). What makes smartphone use meaningful or meaningless? in Proceedings of the ACM on Interactive, Mobile, Wearable and Ubiquitous Technologies, Vol. 2 1–26. 10.1145/3191754

[31] Tossell C Kortum P Shepard C Rahmati A Zhong L Exploring smartphone addiction: Insights from long-term telemetric behavioral measures. Int J Interact Mob Technol (2015) 937–43. 10.3991/ijim.v9i2.4300

[32] Polito V Langdon R Barnier AJ Sense of agency across contexts: Insights from schizophrenia and hypnosis. Psychol Consciousness: Theory Res Pract (2015) 2:301–14. 10.1037/cns0000053

[33] Walsh E Oakley DA Halligan PW Mehta MA Deeley Q The functional anatomy and connectivity of thought insertion and alien control of movement. Cortex (2015) 64:380–93. 10.1016/j.cortex.2014.09.012 25438744

[34] Green JP The five factor model of personality and hypnotizability: Little variance in common. Contemp Hypnosis (2004) 21:161–8. 10.1002/ch.303

[35] Nordenstrom BK Council JR Meier BP The “big five” and hypnotic suggestibility. Int J Clin Exp Hypnosis (2002) 50:276–81. 10.1080/00207140208410103 12088333

[36] Shor RE Orne EC (1962). The Harvard Group Scale of Hypnotic Susceptibilty. 10.1080/00207146308409226 13988658

[37] Laurence J-R Perry C Montreal norms for the Harvard Group Scale of Hypnotic Susceptibility, Form A. Int J Clin Exp Hypnosis (1982) 30:167–76. 10.1080/00207148208407381 7085138

[38] Peterson R A meta-analysis of Cronbach’s coefficient alpha. J Consum Res (1994) 21:381–91. 10.1086/209405

[39] Elhai JD Dvorak RD Levine JC Hall BJ Problematic smartphone use: A conceptual overview and systematic review of relations with anxiety and depression psychopathology. J Affect Disord (2017) 207:251–9. 10.1016/j.jad.2016.08.030 27736736

[40] Kwon M Kim D-J Cho H Yang S The smartphone addiction scale: Development and validation of a short version for adolescents. PloS One (2013) 8:e83558. 10.1371/journal.pone.0083558 24391787 PMC3877074

[41] Algina J Keselman HJ Penfield RD An alternative to Cohen’s standardized mean difference effect size: A robust parameter and confidence interval in the two independent groups case. psychol Methods (2005) 10:317–28. 10.1037/1082-989X.10.3.317 16221031

[42] Hilgard ER Hypnotic susceptibility. San Diego: Harcourt (1965).

[43] Elhai JD Tiamiyu M Weeks J Depression and social anxiety in relation to problematic smartphone use. Internet Res (2018) 28:315–32. 10.1108/intr-01-2017-0019

[44] Cumming G The new statistics: Why and how. Psychol Sci (2014) 25:7–29. 10.1177/0956797613504966 24220629

[45] Schönbrodt FD Perugini M At what sample size do correlations stabilize? J Res Pers (2013) 47:609–12. 10.1016/j.jrp.2013.05.009

[46] Lifshitz M Sheiner EO Olson JA Thériault R Raz A On suggestibility and placebo: A follow-up study. Am J Clin Hypnosis (2017) 59:385–92. 10.1080/00029157.2016.1225252 28300519

[47] Lopez-Fernandez O Short version of the Smartphone Addiction Scale adapted to Spanish and French: Towards a cross-cultural research in problematic mobile phone use. Addictive Behav (2017) 64:275–80. 10.1016/j.addbeh.2015.11.013 26685805

[48] Duke É Montag C Smartphone addiction, daily interruptions and self-reported productivity. Addictive Behav Rep (2017) 6:90–5. 10.1016/j.abrep.2017.07.002 PMC5800562 29450241

[49] Haug S Castro RP Kwon M Filler A Kowatsch T Schaub MP Smartphone use and smartphone addiction among young people in Switzerland. J Behav Addict (2015) 4:299–307. 10.1556/2006.4.2015.037 26690625 PMC4712764

[50] Cocoradă E Maican CI Cazan A-M Maican MA Assessing the smartphone addiction risk and its associations with personality traits among adolescents. Children Youth Serv Rev (2018) 93:345–54. 10.1016/j.childyouth.2018.08.006

[51] Wolniewicz CA Tiamiyu MF Weeks JW Elhai JD Problematic smartphone use and relations with negative affect, fear of missing out, and fear of negative and positive evaluation. Psychiatry Res (2018) 262:618–23. 10.1016/j.psychres.2017.09.058 28982630

[52] Yıldız MA Emotion regulation strategies as predictors of internet addiction and smartphone addiction in adolescents. J Educ Sci Psychol (2017) 7:66–78.

[53] Wang P Zhao M Wang X Xie X Wang Y Lei L Peer relationship and adolescent smartphone addiction: The mediating role of self-esteem and the moderating role of the need to belong. J Behav Addict (2017) 6:708–17. 10.1556/2006.6.2017.079 PMC6034960 29254360

[54] Piccione C Hilgard ER Zimbardo PG On the degree of stability of measured hypnotizability over a 25-year period. J Pers Soc Psychol (1989) 56:289. 10.1037/0022-3514.56.2.289 2926631

[55] Cardeña E The domain of dissociation. In: Lynn SJ Rhue JW, editors. Dissociation: Clinical and theoretical perspectives. New York: Guilford (1994) p. 15–31.

[56] Cardeña E Van Duijl M Weiner L Terhune D (2009). Possession/trance phenomena. In Dell PF, O’Neil JA, editors, Dissociation and the dissociative disorders: DSM-v and beyond. New York: Routledge (2009) 171–81.

[57] Bernardi S Pallanti S Internet addiction: A descriptive clinical study focusing on comorbidities and dissociative symptoms. Compr Psychiatry (2009) 50:510–6. 10.1016/j.comppsych.2008.11.011 19840588

[58] Terhune DB Cardeña E Lindgren M Dissociative tendencies and individual differences in high hypnotic suggestibility. Cogn Neuropsychiatry (2011) 16:113–35. 10.1080/13546805.2010.503048 20721761

[59] Dienes Z Brown E Hutton S Kirsch I Mazzoni G Wright DB Hypnotic suggestibility, cognitive inhibition, and dissociation. Consciousness Cogn (2009) 18:837–47. 10.1016/j.concog.2009.07.009 19709904

[60] Lynn SJ Kirsch I Hallquist MN Social cognitive theories of hypnosis. In: Nash M Barnier AJ, editors. The Oxford handbook of hypnosis: Theory, research, and practice. Oxford University Press (2008) p. 111–39.

[61] Wickramasekera IE II Szlyk JP Could empathy be a predictor of hypnotic ability? Int J Clin Exp Hypnosis (2003) 51:390–9. 10.1076/iceh.51.4.390.16413 14594187

[62] Wickramasekera IE II Mysteries of hypnosis and the self are revealed by the psychology and neuroscience of empathy. Am J Clin Hypnosis (2015) 57:330–48. 10.1080/00029157.2014.978495 25928682

[63] van Deursen AJAM Bolle CL Hegner SM Kommers PAM Modeling habitual and addictive smartphone behavior. Comput Hum Behav (2015) 45:411–20. 10.1016/j.chb.2014.12.039

[64] Veissière SPL Stendel M Hypernatural monitoring: A social rehearsal account of smartphone addiction. Front Psychol (2018) 9:141. 10.3389/fpsyg.2018.01118 29515480 PMC5826267

[65] Fogg BJ Tiny habits: The small changes that change everything. Boston, MA: Houghton Mifflin Harcourt (2019).

[66] Wilmer HH Sherman LE Chein JM Smartphones and cognition: A review of research exploring the links between mobile technology habits and cognitive functioning. Front Psychol (2017) 81–16. 10.3389/fpsyg.2017.00605 PMC5403814 28487665

[67] Fitz N Kushlev K Jagannathan R Lewis T Paliwal D Ariely D Batching smartphone notifications can improve well-being. Comput Hum Behav (2019) 101:84–94. 10.1016/j.chb.2019.07.016

[68] Wu T The attention merchants: The epic scramble to get inside our heads. New York: Vintage (2017).

